# Predictors of willingness of HPV vaccine uptake across Eight States in Nigeria

**DOI:** 10.1186/s12889-025-22000-2

**Published:** 2025-02-24

**Authors:** Hilary I. Okagbue, Grace Erekosima, Sidney Sampson, Brian Atuhaire, Olugbemisola Samuel, Bright Chimezie, Mahfus Dauda, Akolade Jimoh, Gideon Ogbu, Joshua Ayuba, Iveren Shinshima

**Affiliations:** 1 Sydani Institute for Research and Innovation, Sydani Group, Abuja, Nigeria; 2 Department of Mathematics, Covenant University, Ota, Nigeria; 3 Sydani Initiative for International Development, Sydani Group, Abuja, Nigeria; 4 The Vaccine Alliance, Geneva, Switzerland

**Keywords:** Awareness, HPV, HPV vaccine, Regression, Severity, Survey, Susceptibility

## Abstract

**Background:**

The Nigerian Federal Government planned to launch the Human Papillomavirus (HPV) vaccine on September 25, 2023. We therefore aimed to assess caregiver awareness and willingness regarding HPV vaccine uptake for girls aged 9–14 across eight states (Abia, Adamawa, Bayelsa, Benue, Enugu, FCT, Jigawa, and Taraba), evaluating public readiness for the vaccine rollout.

**Methods:**

A cross-sectional telephone survey was conducted in the eight states using a structured questionnaire. Stratified random sampling was employed to ensure representation from each state’s three senatorial districts. Adult participants’ socio-demographic characteristics—including their relationship to the child, gender, settlement type (rural/urban), and age group—were analyzed. Local Government Area and Senatorial Zone were used solely for geographical representation. Awareness, perceived severity, susceptibility, and willingness for HPV vaccine uptake were calculated based on questionnaire responses. Descriptive statistics, correlation analyses, ordinal and linear regression, and mediation regression were applied to derive results.

**Results:**

Our findings indicated low general HPV awareness but high willingness for vaccine uptake among caregivers. Northern states exhibited higher awareness, perceived severity, susceptibility, and willingness compared to Southern states. Significant differences emerged across states in awareness and willingness, with positive correlations observed among awareness, severity, susceptibility, and willingness. Multiple linear regression revealed that awareness does not directly predict willingness, while mediation regression demonstrated that awareness indirectly influences willingness through severity and susceptibility. The study’s implications were analyzed using the Health Belief Model (HBM).

**Conclusion:**

Our survey found geopolitical disparities in HPV awareness and willingness across Nigeria. Future interventions should prioritize emphasizing the severity and susceptibility of HPV-related diseases, particularly in low-resource settings. Providing accurate information from trusted sources and addressing misconceptions through evidence-based strategies can enhance informed decision-making regarding HPV vaccination.

## Background

Human Papillomavirus (HPV), the primary cause of several cancers, is a major contributor to the global prevalence of cervical cancer [1]. The World Health Organization (WHO) identifies high-risk HPV types 16 and 18 as responsible for most HPV-related cancers, including cervical, penile, anal, vulvar, vaginal, oropharyngeal, and oral cancers [2, 3]. HPV, a sexually transmitted infection (STI), spreads through genital skin-to-skin contact and is closely linked to cervical cancer, the second most common cancer among Nigerian women aged 15–44 [4]. Transmission can occur via vaginal, oral, or anal sexual activity.

A 2017 report [5] ranked Nigeria among sub-Saharan African countries with high rates of HPV-related diseases. Cervical cancer is the second-leading cause of cancer mortality in Nigeria, surpassed only by breast cancer [6], with detailed prevalence and mortality data available in [7]. Studies report varying HIV prevalence and seroprevalence rates among Nigerian women [8–10].

To combat rising cervical cancer rates globally, vaccination of girls aged 9–14 is recommended as the optimal preventive strategy against HPV, genital warts, and cervical cancer, particularly in low- and middle-income countries [11].

The Nigerian Federal Government plans to launch the HPV vaccine on September 25, 2023. To gauge public readiness, this study assessed caregiver awareness and willingness regarding HPV vaccine uptake for girls aged 9–14 across eight states: Abia, Adamawa, Bayelsa, Benue, Enugu, FCT, Jigawa, and Taraba. The goal was to evaluate caregivers’ awareness of HPV infection, their knowledge of the vaccine rollout, and their willingness to consent to their children’s vaccination.

Our survey may provide insights into predictors of caregivers’ willingness to vaccinate, including their perceived awareness, severity, and susceptibility of HPV-related risks. Our findings may inform data-driven strategies to promote vaccine uptake, address misconceptions, and reduce barriers to immunization.

We sought to identify predictors of willingness in the uptake of the HPV vaccine by analyzing data from a public opinion survey, aiming to assess public readiness for the vaccine rollout through caregiver awareness and consent for their daughters’ vaccination. Our analytical approach diverges from existing literature [12–15] in three key ways. First, awareness of HPV and its vaccine are combined into a single variable, unlike prior studies [16–19] that treated them separately, given their significant positive correlation and redundancy when analyzed apart. Second, demographic variables were omitted from the predictive model to enhance its adaptability for broader application. Third, the analysis was conducted at the state level without aggregating results, enabling granular insights into regional patterns. Our state-specific focus serves two purposes: it acknowledges the exclusion of Southwest Nigerian states from the sample and allows tailored visualization of each state’s trends, facilitating targeted strategies to boost HPV vaccine uptake during implementation.

## Materials and methods

### Survey

The survey was conducted across eight states from September 4 to September 15, 2023, targeting a purposive sample of 500 caregivers per state. Data were collected via telephone interviews, with survey quotas allocated to ensure proportional representation of each Local Government Area (LGA). Post-stratification weights for gender and LGA were applied to improve representativeness and align the data with population estimates, adjusted from 2006 census figures to 2022 projections. The margin of error is ± 4.65% at a 95% confidence level, with sample sizes detailed in Table 1. Interviews were administered in respondents’ preferred languages, including Igbo, Hausa, Fulani, Ijaw, Jukun, Tiv, Idoma, English, Pidgin English, and other local languages. All participants were aged 18 years or older. A purpose-designed questionnaire was used, and non-response rates were minimized through strict adherence to the script, with minimal probing.

**Table 1:** The sample size of the surveyed caregivers across 8 states

State	Sample size
Abia	502
Adamawa	514
Bayelsa	518
Benue	506
Enugu	517
FCT	503
Jigawa	507
Taraba	507

The targeted sample size was 4,000 caregivers; however, the final purposive sample comprised 4,074 participants

### Study variables

The variables analyzed encompass participants’ socio-demographic characteristics, including their relationship to the child (father, mother, or guardian), gender (male or female), settlement type (rural or urban), and age group (18–35, 36–60, 61 + , or prefer not to say). Two additional demographic variables namely the Local Government Area (LGA) and Senatorial Zone were excluded from analysis but used to ensure the sample’s geographical diversity.

Three questions were utilized as measures of awareness, and the formula was applied for analysis.

1 $$\text{Awareness}=Mean\left({\sum }_{1}^{n}\left(\text{AHI}+\text{AHV}+\text{AGP}\right)\right) Awareness \in \left\{0.3\right\}$$

The analysis included three binary variables: AHI (awareness of HPV), AHV (awareness of the HPV vaccine), and AGP (awareness of government plans to introduce HPV vaccination). These variables were coded as 0 (“no”) and 1 (“yes”), yielding composite awareness scores ranging from 0 to 3.

Willingness for HPV vaccine uptake was assessed using two questions: respondents’ willingness to consent to their female children receiving the HPV vaccine (RWH) and spouse willingness to consent to their female children’s vaccination (SWH). However, SWH was excluded from combined statistical analysis due to the subjective nature of responses (recorded as “no” or “yes”) and potential biases, such as variability between respondents and spouses or biases among guardians and single parents. Consequently, SWH was not used as a measure of willingness in this study; only its frequency distribution was reported.

Lastly, susceptibility and severity were calculated. Susceptibility pertains to the risk or likelihood of contracting HPV, while severity relates to the seriousness of health outcomes or societal impacts of HPV infection. The two questions used to measure susceptibility were “A girl child is at risk for getting an HPV infection” and “Any girl child is likely to contract an HPV infection in her lifetime.” The mean of the responses to these two questions was used to compute susceptibility and severity, respectively.

### Statistical analysis

The collected data for the study underwent analysis using Statistical Package for Social Sciences (SPSS) version 25.0 for Windows (IBM Corp., Armonk, N.Y., USA) and SPSS Process Macros version 4.2 (Andrew F. Hayes). Descriptive statistics, including frequencies, were calculated for categorical sociodemographic variables. A combination of frequencies and means was utilized for measures of awareness, willingness of HPV vaccine uptake, susceptibility, and severity.

Frequency analysis was employed for the descriptive analysis of sociodemographic variables, awareness, willingness of HPV vaccine uptake, severity, and susceptibility. Bivariate analyses utilized Pearson correlation, while multivariate analyses involved ordinal regression and linear regression. A p-value < 0.05 was considered statistically significant, unless specified otherwise. Additional multivariate analysis was conducted using a mediation regression model with one independent variable, one dependent variable, and two mediators. For selected confirmatory analyses, Kruskal–Wallis, Chi-square test of independence, and analysis of variance (ANOVA) were applied.

## Results

### Sociodemographic characteristics

In all surveyed states, the majority of respondents identified as fathers, followed by guardians and then mothers, as shown in Table 2. Jigawa State stands out with the highest proportion of fathers (74.8%), while Enugu State has the lowest percentage of fathers (32.7%) but the highest proportion of mothers of the young girls. Overall, more males than females were surveyed via telephone interviews. Most respondents resided in urban areas and fell into the 36–60 age group. As anticipated, a majority had completed secondary or tertiary education, partly due to their urban residence. Notably, rural–urban migration remains prevalent in Nigeria [20]**,** with a larger population concentrated in urban areas. Consequently, surveys are more likely to include participants from urban rather than rural settings.

**Table 2:** Sociodemographic summary

	Abia (%)	Adamawa (%)	Bayelsa (%)	Benue (%)	Enugu (%)	FCT (%)	Jigawa (%)	Taraba (%)
**Relationship with child**								
Father	263 (52.4)	280 (54.5)	223 (43.0)	250 (49.5)	169 (32.7)	201 (40.0)	379 (74.8)	219 (43.2)
Mother	128 (25.5)	73 (14.2)	160 (30.9)	66 (13.0)	168 (32.5)	167 (33.2)	34 (6.7)	88 (17.4)
Guardian	111 (22.1)	161 (31.3)	135 (26.1)	190 (37.5)	180 (34.8)	135 (26.8)	84 (18.5)	200 (39.4)
**Gender**								
Male	327 (65.1)	392 (76.3)	283 (54.6)	395 (78.1)	268 (51.8)	267 (53.1)	464 (91.5)	343 (67.7)
Female	175 (34.9)	122 (23.7)	235 (45.4)	111 (21.9)	249 (48.2)	236 (46.9)	43 (8.5)	164 (32.3)
**Settlement**								
Rural	141 (28.1)	233 (45.3)	190 (36.7)	159 (31.4)	150 (29.0)	118 (23.5)	224 (44.2)	212 (41.8)
Urban	361 (71.9)	281 (54.7)	328 (63.3)	347 (68.6)	367 (71.0)	385 (76.5)	283 (55.8)	295 (58.2)
**Age Group**								
18–35	61 (12.2)	146 (28.4)	86 (16.6)	133 (26.3)	142 (27.5)	99 (19.7)	86 (17.0)	146 (28.8)
36–60	376 (74.9)	333 (64.8)	397 (76.6)	341 (67.3)	311 (60.2)	372 (74.0)	383 (75.5)	332 (65.4)
61 +	29 (5.8)	28 (5.4)	15 (2.9)	14 (2.8)	27 (5.2)	10 (1.9)	32 (6.3)	16 (3.2)
Prefer not to say	36 (7.2)	7 (1.4)	20 (3.9)	18 (3.6)	37 (7.1)	22 (4.4)	6 (1.2)	13 (2.6)
**Education**								
No formal /primary	41 (8.2)	41 (8.0)	26 (5.0)	31 (6.1)	51 (9.9)	24 (4.7)	71 (14.0)	44 (8.7)
Secondary	184 (36.7)	140 (27.2)	153 (29.5)	132 (26.1)	181 (35.0)	119 (23.7)	117 (23.1)	129 (25.4)
Tertiary	235 (46.8)	320 (62.3)	312 (60.2)	323 (63.8)	249 (48.1)	336 (66.8)	316 (62.3)	325 (64.1)
Prefer not to say	42 (8.4)	13 (2.5)	27 (5.2)	20 (4.0)	36 (7.0)	24 (4.8)	3 (0.6)	9 (1.8)

### General level of awareness

Awareness of HPV is highest in Jigawa State (34.3%) and lowest in Enugu State (19.7%), while awareness of the HPV vaccine is greatest in Jigawa State (29.6%) and lowest in Benue State (18.2%), as detailed in Table 3. Similarly, knowledge of the government’s plans to introduce HPV vaccination is most prevalent in Jigawa State (34.3%) and least common in Enugu State (19.7%).

**Table 3:** Summary of respondents answered yes on the 3 questions

State	N	AHI	AHV	AGP	Awareness
Abia	502	124 (24.7%)	133 (26.5%)	100 (19.9%)	0.71 (23.7%)
Adamawa	514	167 (32.5%)	117 (22.8%)	160 (31.1%)	0.86 (28.7%)
Bayelsa	518	112 (21.6%)	117 (22.6%)	86 (16.6%)	0.61 (20.3%)
Benue	506	101 (20.0%)	92 (18.2%)	99 (19.6%)	0.58 (19.3%)
Enugu	517	102 (19.7%)	103 (19.9%)	67 (13.0%)	0.52 (17.3%)
FCT	503	140 (27.8%)	137 (27.2%)	125 (24.9%)	0.80 (26.7%)
Jigawa	507	174 (34.3%)	150 (29.6%)	195 (38.5%)	1.02 (34.0%
Taraba	507	143 (28.2%)	112 (22.1%)	141 (27.8%)	0.78 (26.0%)

*AHI *awareness of HPV infection, *AHV *awareness of the HPV vaccine, *AGP *awareness of government plans to introduce HPV vaccination

Overall awareness remains relatively low, with the lowest levels in Enugu State (17.3%) and the highest in Jigawa State (34.0%). Northern states exhibit higher awareness compared to Southern states, a regional disparity confirmed by the Kruskal–Wallis test, which revealed statistically significant differences in median awareness across the eight states (*H* = 107.702, *p* < 0.001, *df* = 7).

### Willingness of HPV vaccine uptake

Two variables were used to assess willingness to receive HPV vaccination. Respondents’ willingness to consent to their daughters receiving the HPV vaccine (RWH) was highest in Jigawa State (4.58/5.00) and lowest in Bayelsa State (4.05/5.00). Northern Nigerian states demonstrated greater willingness to accept HPV vaccination compared to Southern states, as shown in Table 4.

**Table 4:** Summary of measures of willingness of HPV vaccine uptake

State	Mean RWH	SWH	Chi-square test
Abia	4.08	371 (73.9%)	140.94**
Adamawa	4.47	454 (88.3%)	143.58**
Bayelsa	4.06	380 (73.4%)	189.24**
Benue	4.09	395 (78.1%)	169.33**
Enugu	4.13	376 (72.7%)	220.00**
FCT	4.13	387 (76.9%)	205.05**
Jigawa	4.58	489 (96.4%)	284.00**
Taraba	4.44	445 (87.8%)	204.91**

*RWH *Respondents’ willingness to consent to their female children receiving the HPV vaccine, *SWH *spouse willingness to consent to their female children’s vaccination

*p value less than 0.05

**p value less than 0.001

Similarly, spouse willingness (SWH) was excluded from further analysis due to its subjective nature and challenges in integrating it with RWH, given their distinct measurement scales. Overall, most respondents reported that their spouses were willing to allow their children to receive the HPV vaccine. A Chi-square test confirmed a statistically significant association between RWH and SWH across the eight states studied.

The Kruskal–Wallis test revealed significant median differences in willingness (RWH) across the eight states (*H* = 257.121, *p* < 0.001, *df* = 7).

### Measures of risk

Severity and susceptibility were utilized as measures of risk. In this context, *severity* refers to the perceived seriousness or health impact of HPV infection, while *susceptibility* reflects an individual’s perceived likelihood of contracting HPV without vaccination. As summarized in Table 5, risk patterns aligned with trends in awareness and willingness: Northern Nigerian states exhibited higher mean severity and susceptibility scores compared to Southern states. Bayelsa State recorded the lowest scores for both risk measures, whereas Jigawa State ranked highest.

**Table 5:** Summary of measure of risk

State	Mean susceptibility	Mean severity
Abia	3.69	3.78
Adamawa	4.04	4.03
Bayelsa	3.56	3.67
Benue	3.76	3.83
Enugu	3.66	3.77
FCT	3.76	3.85
Jigawa	4.12	4.19
Taraba	3.97	4.02

Two separate one-way ANOVA tests were conducted for the two risk measures. The first test revealed a significant mean susceptibility difference across the eight states (*F* = 43.863, *p* < 0.001). The second test similarly identified a significant mean severity difference across the eight states (*F* = 33.562, *p* < 0.001).

#### Measure of relationships

Spearman’s rank correlation was used to assess correlations between the four variables—awareness, willingness, susceptibility, and severity—with results summarized in Table 6.

**Table 6:** Summary of correlation results

State	Awareness vs willingness	Awareness vs susceptibility	Awareness vs severity	willingness vs susceptibility	willingness vs severity	susceptibility vs severity
Abia	0.156**	0.200**	0.196**	0.224**	0.257**	0.641**
Adamawa	0.065	0.153**	0.084	0.210**	0.248**	0.648**
Bayelsa	0.082	0.109*	0.139*	0.310**	0.319**	0.686**
Benue	0.084	0.177**	0.149*	0.379**	0.317**	0.652**
Enugu	0.098*	0.143*	0.194**	0.287**	0.291**	0.593**
FCT	0.065	0.181**	0.143*	0.420**	0.306**	0.692**
Jigawa	−0.017	0.181**	0.137*	0.396**	0.322**	0.671**
Taraba	0.196**	0.216**	0.238**	0.328**	0.350**	0.649**

^*^p value less than 0.05

**p value less than 0.001

**Fig. 1:**
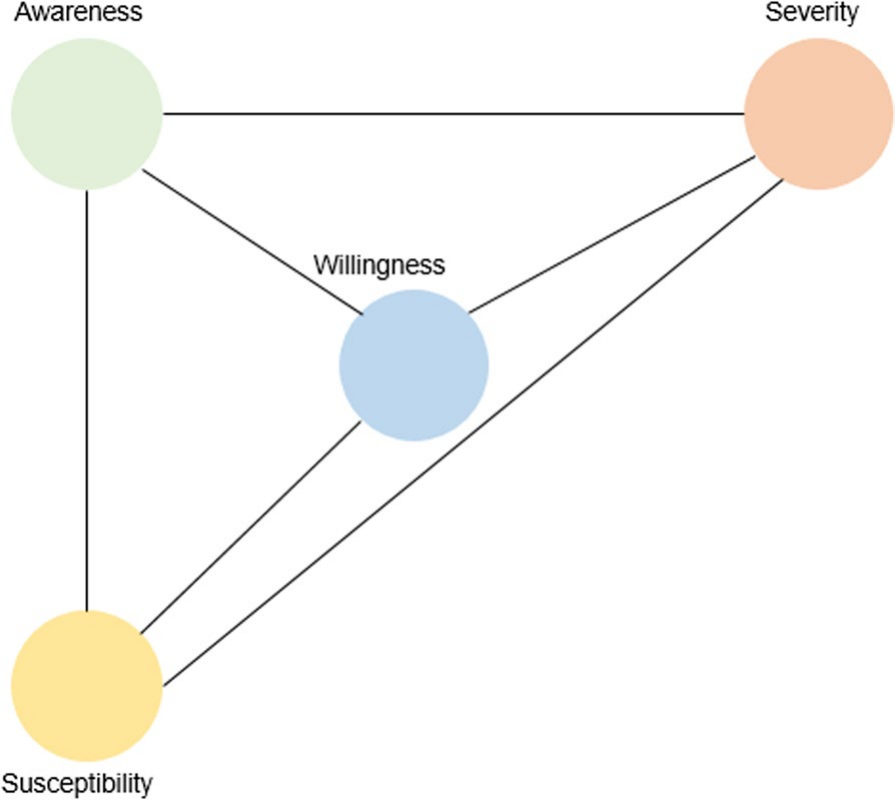
The correlation network

Positive correlations were consistently observed, except for a non-significant negative correlation between awareness and willingness in Jigawa State. Only 3 out of 8 correlations between awareness and willingness were statistically significant. All but one correlation between awareness and severity was significant. Notably, significant positive correlations were identified for the following pairs: awareness and susceptibility, willingness and susceptibility, willingness and severity, and severity and susceptibility. These relationships are visualized in a correlation network graph (Fig. 1), which illustrates consistent positive associations across all variables.

#### Predictors of willingness of HPV vaccine uptake

Since only one variable was used to measure willingness, two regression analyses were conducted to predict HPV vaccine uptake willingness, using awareness, susceptibility, and severity. Ordinal regression was applied due to the ordinal nature of the willingness measure (see Table 7), while multiple linear regression (MLR) served as a precursor to mediation analysis.

**Table 7:** Predictors of willingness (Ordinal Regression)

		Evaluation			Coefficient		Diagnostics
State	−2 Log Likelihood	Deviance	Nagelkerke	Awareness	Susceptibility	Severity	TOPL (Chi-square)
Abia	488.309**	347.769	0.106	0.283*	0.267	0.540*	14.304
Adamawa	417.561**	282.061	0.095	0.089	0.322*	0.600**	15.351
Bayelsa	437.824**	273.75	0.133	0.133	0.483*	0.635**	16.33
Benue	384.512**	257.358	0.204	0.029	1.035**	0.573*	14.901
Enugu	453.840**	306.461	0.121	0.151	0.552**	0.548*	17.803*
FCT	425.626**	286.229	0.224	0.014	1.264**	0.320	9.453
Jigawa	293.650**	206.472	0.218	−0.186*	1.264**	0.529*	16.436*
Taraba	340.730**	233.872	0.208	0.299*	0.614*	0.822**	13.843

*TOPL *Test of Parallel lines

*p value less than 0.05

**p value less than 0.001

The ordinal regression analysis indicated statistically significant models. However, non-significant deviance values suggested adequate model fit despite low Nagelkerke pseudo R^2^ values. The parallel lines test was significant in Enugu and Jigawa states, warranting cautious interpretation of ordinal regression results.

Awareness emerged as a significant predictor only in Abia, Jigawa, and Taraba states, whereas susceptibility significantly predicted willingness in all states except Abia. Similarly, severity proved significant in all states except the FCT.

The MLR results presented in Table 8 indicate statistically significant regression models, with coefficients of determination ranging from 6.9% to 17.7%. Notably, only results from Adamawa State passed the rigorous Breusch-Pagan heteroscedasticity test. However, the models passed multicollinearity tests, as evidenced by tolerance values and Variance Inflation Factor (VIF), confirming no significant correlations among the three independent variables.

**Table 8:** Predictors of willingness (MLR)

	Evaluation			Coefficient				Diagnostics	
State	R Square	F	Constant	Awareness	Susceptibility	Severity	AVIF	Average Tolerance	Breusch-Pagan
Abia	0.082	14.854**	2.717	0.090*	0.117	0.229*	1.484	0.712	9.036*
Adamawa	0.069	12.593**	3.298	0.043	0.076	0.206*	1.498	0.71	6.682
Bayelsa	0.123	23.947**	2.130	0.079	0.248*	0.272*	1.604	0.679	12.432*
Benue	0.153	30.231**	2.078	0.030	0.373**	0.156*	1.512	0.705	15.180*
Enugu	0.109	20.921**	2.458	0.064	0.210*	0.232*	1.38	0.752	24.816**
FCT	0.177	35.749**	2.145	0.005	0.490**	0.037	1.629	0.669	15.593*
Jigawa	0.170	34.371**	2.895	−0.047*	0.324**	0.095	1.564	0.689	35.997**
Taraba	0.152	30.119**	2.813	0.079*	0.163*	0.228**	1.52	0.694	21.801**

*AVIF *Average variance inflation factor

*p value less than 0.05

**p value less than 0.001

#### Mediation models

**Fig. 2:**
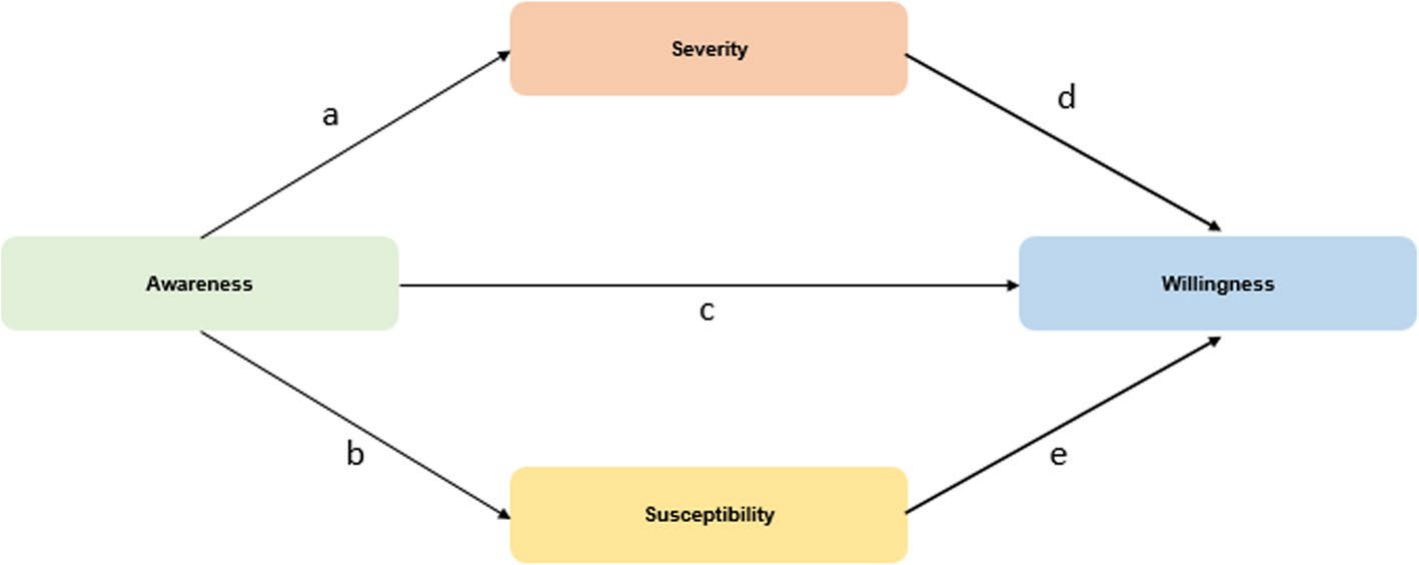
The mediation conceptual framework

The final regression model predicts willingness using awareness as the independent variable, with severity and susceptibility acting as mediators. A mediation analysis was conducted to examine the indirect effect of awareness on willingness through the two mediator variables (severity and susceptibility). This conceptual framework is depicted in Fig. 2.

The conceptual framework outlined five distinct relationships, labeled as a, b, c, d, and e. Specifically, awareness influences severity (a), which in turn impacts willingness (d). Similarly, awareness affects susceptibility (b), which subsequently influences willingness (e). A direct relationship between awareness and willingness is represented by (c). The parameter values for these relationships across the eight states were derived through mediation analysis, and a summary of the results is shown in Table 9.

**Table 9:** Mediation models summary

State	a	b	c	d	e
Abia	0.120**	0.108**	0.090*	0.229*	0.117
Adamawa	0.063*	0.110**	0.043	0.206**	0.076
Bayelsa	0.099*	0.068*	0.079	0.272**	0.248*
Benue	0.084*	0.127**	0.030	0.156*	0.373**
Enugu	0.128**	0.114*	0.064	0.231**	0.210**
FCT	0.078*	0.105**	0.005	0.037	0.490**
Jigawa	0.061*	0.090**	−0.047*	0.095	0.324**
Taraba	0.152**	0.150**	0.079*	0.228**	0.163*

^*^p value less than 0.05

**p value less than 0.001

The mediation analysis summary indicates that awareness positively influences severity, which in turn enhances willingness. Similarly, awareness has a positive effect on susceptibility, which subsequently affects willingness. Notably, only three states show a significant direct relationship between awareness and willingness, with one of these displaying a *negative* association. No negative influences were observed in the remaining states.

## Discussion

### General findings

Our study assessed caregiver awareness and willingness toward the Nigerian Federal Government’s planned HPV vaccine rollout for girls aged 9–14, targeting eight states (Abia, Adamawa, Bayelsa, Benue, Enugu, FCT, Jigawa, Taraba) through a cross-sectional telephone survey employing stratified random sampling across senatorial districts. Using structured questionnaires, socio-demographic variables (e.g., caregiver-child relationship, gender, settlement type, age) were analyzed alongside awareness (derived from knowledge of HPV, its vaccine, and government plans) and willingness to vaccinate, with statistical methods including descriptive analyses, regression, and mediation models.

Our study surveyed more males than females, a finding consistent with prior research indicating higher mobile phone ownership among males in Nigeria, particularly in rural areas [21, 22]. However, the results may vary if female participation increases, as females often possess greater insight into their daughters’ sexual health [23]. This dynamic is complicated by Nigeria’s patriarchal societal structure, where men typically assume decision-making roles within families [24].

The low HPV awareness reported here contrasts with findings from HPV vaccination campaigns. For instance, caregivers in Ogun State demonstrated high awareness during a campaign conducted from 24 to 28th October 2023 [25], likely due to the successful implementation of Advocacy, Communication, and Social Mobilization (ACSM) strategies [26]. This study reinforces evidence that ACSM-driven campaigns significantly improve HPV vaccine awareness and demand in Nigeria [27]. It also aligns with pre-campaign research [12–16] showing low caregiver awareness of HPV, HPV vaccines, and willingness to consent to vaccination. Examples include cross-sectional studies in Kano State (December 2022) [28] and Plateau State (2019) [29], which reported similarly low baseline metrics.

Discrepancies in awareness levels are evident when comparing findings across studies. In Benue State, previous research involving secondary schoolgirls reported awareness levels of 15.8%–22.5% [30], compared to 19.3% in our study. Similarly, Enugu State studies reported varying rates: 13.86% [31], 41% [32], 26%–43.5% [33], and 55.3%–58.5% [34], differing from the 17.3% recorded here. Notably, these studies involved distinct populations (secondary schoolgirls, caregivers, and mothers), highlighting the influence of respondent demographics.

Northern states exhibited higher HPV awareness, willingness for vaccination, perceived severity, and susceptibility compared to Southern states. Statistical analyses revealed significant median and mean differences in these variables across the eight states, underscoring a geopolitical divide. This suggests that interventions to improve HPV vaccine uptake must be regionally tailored, as uniform national strategies have proven ineffective. Future efforts should prioritize identifying context-specific barriers to awareness and uptake, alongside addressing sociodemographic factors [35]. Our findings align with proposals for evidence-based interventions [36], including targeted research to address regional disparities. Implementing such measures could prevent HPV from exacerbating Nigeria’s existing vaccination inequities and persistent zero-dose communities [37].

Our study reveals several findings consistent with existing literature. First, awareness demonstrates a positive correlation with HPV vaccine uptake [38], suggesting that caregivers with greater knowledge of HPV and its vaccine are more likely to consent to vaccination for their wards. Higher awareness of HPV infection is thus associated with increased vaccination likelihood [39]. Second, awareness correlates positively with perceived susceptibility [40], indicating that informed caregivers are more likely to view their wards as vulnerable to HPV infection. Third, awareness of HPV infection is positively linked to perceived severity [41], implying that caregivers with adequate HPV knowledge are more likely to recognize the infection’s potential gravity. Consequently, well-informed caregivers are better positioned to act preemptively.

Fourth, perceived susceptibility positively correlates with willingness for HPV vaccination [42], meaning caregivers who perceive their wards as at risk are more inclined to vaccinate. This contrasts with a recent Nigerian study reporting a weak negative correlation between susceptibility perception and vaccination willingness (*r* = −0.06, *p* = 0.215) [3], a discrepancy potentially attributable to the latter’s homogeneous sample of private university students. Fifth, the perceived severity of HPV infection positively influences vaccination willingness [43], as caregivers who view HPV as serious are more motivated to act. This aligns with research showing that perceived consequences drive vaccination decisions [44]. Finally, perceived susceptibility and severity are interrelated: caregivers who deem their wards vulnerable to HPV are also more likely to perceive the infection as severe.

Transitioning to regression analysis, both ordinal and multiple linear regression models identify perceived susceptibility and severity as significant predictors of vaccination willingness. Notably, awareness alone does not directly increase HPV vaccine uptake [45]; knowledge of HPV does not inherently translate to vaccination intent [46]. Thus, susceptibility and severity perceptions outweigh awareness as predictors, implying caregivers prioritize vaccination when they perceive HPV as both a personal threat and a severe health risk—particularly given HPV’s established link to cervical cancer. Conversely, mere awareness may fail to overcome barriers like misinformation [47] or negative vaccination experiences [48]. While awareness highlights the threat, susceptibility personalizes it, and severity underscores the urgency of action.

The mediation analysis indicates that awareness positively influences susceptibility and severity perceptions, which subsequently enhance vaccination willingness. This suggests that caregivers informed through trusted channels may recognize both their wards’ vulnerability and HPV’s potential consequences, thereby increasing their inclination to vaccinate—a finding supported by studies emphasizing caregivers’ role in adolescent vaccination decisions [49]. Consequently, public health campaigns should not only disseminate HPV information but also address susceptibility and severity perceptions, particularly in low-resource settings. Evidence-based strategies to counter misinformation and leverage trusted sources could improve informed decision-making. These insights align with a structural equation model from India, where awareness, severity, and susceptibility shaped parental vaccination consent [50].

### Health Belief Model (HBM)

The findings of our work align with the constructs of the Health Belief Model (HBM), which explains health-related behaviors based on individuals’ perceptions of health threats and benefits of preventive actions [51]. The results indicate that awareness of HPV and its vaccine is positively correlated with willingness for vaccine uptake, but awareness alone does not directly predict willingness. This suggests that awareness functions primarily as an enabling factor, influencing willingness through perceived severity and susceptibility. In this context, caregivers or parents who are aware of HPV and the vaccine are more likely to recognize the severity of HPV-related diseases and perceive their wards as susceptible, thereby increasing their willingness to vaccinate. The mediation analysis supports this, demonstrating that awareness indirectly influences willingness through these constructs, reinforcing the HBM’s assertion that perceived severity and susceptibility are key determinants of health-related behaviors [52].

The geopolitical variations observed in awareness, severity, susceptibility, and willingness further highlighted the role of sociocultural and structural factors in shaping health perceptions. The higher levels of awareness and willingness in Northern states, compared to the South, suggest that regional differences in public health messaging, cultural beliefs, and access to information play a significant role [53]. This aligns with HBM’s emphasis on modifying factors, such as demographic and sociocultural influences, that shape individuals' health beliefs and actions especially in Nigeria that is a conglomerate of diverse culture, religion and ethnicities. The paper's findings also reveal that merely increasing awareness without addressing perceptions of severity and susceptibility may not significantly drive HPV vaccine uptake. These psychosocial barriers (perceptions of severity and susceptibility) must be eliminated to drive HPV vaccine uptake [54]. This is particularly relevant in contexts where caregivers may have knowledge of HPV but do not see it as an immediate threat to their children, reducing their urgency to act.

The positive correlation between perceived susceptibility and willingness suggests that caregivers who recognize their wards as at risk for HPV infection are more likely to consent to vaccination. This aligns with HBM’s notion that individuals are more likely to engage in preventive behaviors when they perceive themselves or their dependents as vulnerable to a health threat [55]. The significant role of perceived severity further strengthens this framework, as caregivers or parents who believe that HPV infection has severe consequences are more likely to take preventive action. The findings indicate that severity and susceptibility are stronger predictors of willingness than awareness alone, demonstrating that knowledge without a sense of urgency or risk perception may not be sufficient to drive HPV vaccine uptake. Knowledge alone could perpetuate vaccine hesitancy without considering psychosocial variables [56].

Our results also highlight the importance of strategic health communication and intervention approaches. Given that awareness is a precursor to perceived susceptibility and severity, interventions should not only focus on disseminating information about HPV but also on framing messages that enhance the perception of risk and highlight the serious consequences of HPV-related diseases (evidence-based messaging), notably cervical cancer [57]. This aligns with previous research emphasizing that risk perception influences vaccine acceptance more than awareness alone [58]. Our findings further suggest that addressing misconceptions and providing accurate information from trusted sources can strengthen the perceived severity and susceptibility of HPV infection [59], thereby enhancing willingness for vaccination, especially for caregivers and parents from poor educational background.

Our study’s mediation analysis further supports the HBM by illustrating how awareness serves as a foundational factor that influences willingness through susceptibility and severity. This aligns with studies indicating that health communication campaigns are most effective when they integrate elements that enhance perceived risk and emphasize the benefits of preventive behaviors [26, 27, 60]. The observed positive correlation between susceptibility and severity in this paper suggests that caregivers who acknowledge the vulnerability of their wards also tend to perceive HPV as a serious health risk, reinforcing the interdependence of these constructs in shaping health behaviors. Public health interventions should therefore incorporate evidence-based strategies that emphasize both the likelihood of HPV infection and its potential consequences to drive behavioral change which are part of policy recommendations from a recent study [61].

### Sustainable development goals

Our study supports SDG 3 (Good Health and Well-being), SDG 5 (Gender Equality), and SDG 10 (Reduced Inequalities) by addressing barriers to HPV vaccination for girls in Nigeria. It highlights the role of caregiver awareness in vaccine uptake, which is crucial for cervical cancer prevention. Cervical cancer remains a leading cause of death among Nigerian women [62]. Our study submitted how low HPV awareness and the impact of perceived susceptibility and severity on vaccine willingness, could provide insights on how to improve vaccination rates (SDG 3.4). Regional disparities in awareness and willingness between Northern and Southern states further stress the need for context-specific interventions, promoting equitable health access (SDG 10).

Our findings calls for targeted public health messaging to improve HPV risk awareness, aligning with SDG 3.7 (sexual and reproductive health) and SDG 4.7 (health education). By integrating cultural and sociodemographic factors, the study supports Nigeria’s efforts to enhance health literacy and vaccine equity. The mediation analysis further highlights how awareness influences vaccine willingness, providing a framework for effective, sustainable interventions that strengthen Nigeria’s HPV vaccination program and broader health policies such as expanded program on immunization (EPI) and routine immunization (RI).

### Limitations

The use of telephone interviews for data collection presents several limitations. Sampling bias is inherent in telephone-based data collection due to reliance on telephone access, excluding individuals without telephones and potentially skewing representativeness. In contexts like Nigeria, where socioreligious norms are prominent, respondents may withhold sensitive information (e.g., marital or parental status), leading to inclusion bias; face-to-face interviews could mitigate this by fostering trust and enabling accurate participant screening. However, resource constraints necessitated the use of telephone interviews.

Interviewer bias is another concern, as an interviewer’s tone, vocal cues, or limited HPV-related knowledge may inadvertently influence responses. To address this, interviewers received comprehensive training and were selected for their proficiency in respondents’ local languages. Technical challenges, such as poor network coverage in certain regions, risked disrupting communication and compromising response accuracy. Interviewers were provided sufficient airtime, and calls were conducted during daytime hours to optimize connectivity.

Finally, quality control limitations arose from the immediate deletion of phone numbers post-interview, a measure implemented to ensure confidentiality and prevent post-study contact. While necessary, this precluded data verification.

## Conclusion

We used telephone interviews to collect data on HPV awareness, perceived susceptibility, perceived severity, and willingness to uptake the HPV vaccine among caregivers across eight states in Nigeria’s diverse geopolitical regions. Our survey results provided valuable insights into caregivers’ awareness levels, concerns, and willingness regarding HPV vaccination. Notably, awareness, willingness to vaccinate, perceived severity, and susceptibility varied significantly across states, with higher levels observed in Northern states compared to Southern counterparts. The interplay between these variables became clearer when awareness, severity, and susceptibility were used to predict willingness. Furthermore, perceived risk (mediated by susceptibility and severity) positively influenced the relationship between awareness and willingness to vaccinate.

Our research contributes to the field by integrating policy recommendations into the discussion of findings. The conceptual framework derived from mediation analysis can guide the design of interventions to reduce barriers and improve HPV vaccine uptake. Our findings emphasized the need for tailored and targeted public health strategies that extend beyond awareness campaigns to actively address caregivers’ perceptions of HPV’s severity and susceptibility. By leveraging constructs from the HBM, future interventions can enhance HPV vaccine promotion efforts, ensuring caregivers not only receive information but also develop a strong sense of threat perception, thereby increasing vaccination rates.

## Data Availability

The data that support the findings of this study are available from the corresponding author upon reasonable request.

## Abbreviations

HBM: Health Belief Model
HPV: Human Papillomavirus
LGA: Local Government Area
NOI: Ngozi Okonjo Iweala
SDG: Sustainable Development Goal
SRH: Sexual and Reproductive Health
STI: Sexually transmitted infection
WHO: World Health Organization
